# Reduced First-Phase Ejection Fraction and Sustained Myocardial Wall Stress in Hypertensive Patients With Diastolic Dysfunction: A Manifestation of Impaired Shortening Deactivation That Links Systolic to Diastolic Dysfunction and Preserves Systolic Ejection Fraction

**DOI:** 10.1161/HYPERTENSIONAHA.116.08545

**Published:** 2017-03-08

**Authors:** Haotian Gu, Ye Li, Henry Fok, John Simpson, Jonathan C. Kentish, Ajay M. Shah, Philip J. Chowienczyk

**Affiliations:** From the King’s College London, British Heart Foundation Centre, London, United Kingdom (H.G., Y.L., H.F., J.C.K., A.M.S., P.J.C.); and Department of Congenital Heart Disease, Evelina London Children’s Hospital, United Kingdom (J.S.).

**Keywords:** blood pressure, diastole, echocardiography, hypertension, ventricular function

## Abstract

Impaired shortening deactivation of cardiac myocytes could sustain myocardial contraction, preserving ejection fraction at the expense of diastolic dysfunction. We examined the relationship between first-phase ejection fraction (EF1), the fraction of left ventricular volume ejected from the start of systole to the time of the first peak in left ventricular pressure (corresponding to the time of maximal ventricular shortening) to the duration of myocardial contraction and diastolic function in patients with hypertension (n=163), and varying degrees of diastolic dysfunction. Left ventricular systolic pressure was estimated by carotid tonometry; time-resolved left ventricular cavity and wall volume were obtained by echocardiography with speckle wall tracking. Measurements were repeated after nitroglycerin, a drug known to influence ventricular dynamics, in a subsample (n=18) of patients. EF1 and time of onset of ventricular relaxation (as determined from the temporal pattern of myocardial wall stress) were independently correlated with diastolic relaxation as measured by tissue Doppler early diastolic mitral annular velocity (E′, standardized regression coefficients 0.48 and −0.34 for EF1 and time of onset of ventricular relaxation, respectively, each *P*<0.001, irrespective of adjustment for age, sex, antihypertensive treatment, measures of afterload, and ventricular geometry) and with diastolic function measured by the ratio of transmitral Doppler early filling velocity (E) to E′ (E/E′, regression coefficients −0.34 and 0.34, respectively, each *P*<0.001). Nitroglycerin increased EF1, decreased time of onset of ventricular relaxation, and improved diastolic function (each *P*<0.05). Hypertensive patients with diastolic dysfunction exhibit reduced EF1 which may sustain myocardial contraction, preserving systolic ejection fraction at the expense of impaired diastolic function.

## Abstract 2

Supplemental Digital Content is available in the text.

**See Editorial Commentary, pp 575–577**

Isolated muscle, especially cardiac muscle, exhibits shortening deactivation,^1^ whereby shortening of the myocyte after depolarization leads to alterations in the cytosolic calcium transient^2^ and to a smaller and abbreviated contraction compared with that observed under isometric conditions. A corresponding effect has been reported in isolated rabbit hearts.^3^ The molecular basis for shortening deactivation is unclear but is likely to include a sarcomere length-dependent reduction in myofibrillar calcium sensitivity^4^ and a velocity-dependent reduction of myosin crossbridge attachment^5^ that may involve a stress-dependent locking of myosin motors onto the thick filament.^6^ Shortening deactivation could contribute to the rapid reduction in myocardial wall stress (which approximates tension per cross-sectional area in individual myocytes) that, in healthy subjects, occurs early in systole at a time close to that of the first peak in left ventricular (LV) pressure waveform (T1, Figure 1),^7^ which coincides with peak aortic flow^8^ and hence maximum velocity of fiber shortening. This early peak and subsequent fall in myocardial wall stress may facilitate relaxation in diastole. Conversely, an impairment of early-phase shortening velocity and initial ejection would be expected to result in an impairment of deactivation and hence sustain myocardial wall stress to preserve total ejection fraction (EF) at the expense of impaired diastolic function. Close coupling between systolic contraction and relaxation has been shown in integrated animal models and in patients with coronary heart disease and heart failure.^9–12^

We investigated to what degree the shortening deactivation paradigm might account for a relationship between systolic and diastolic function in patients with hypertension in whom some degree of diastolic function is prevalent. To quantify early systolic shortening, we measured first-phase EF (EF1), the EF up to the time of T1. We examined the relationship between EF1, the time of onset of myocardial relaxation (TOR, as measured from the temporal pattern of myocardial wall stress), the ratio of late- to early-phase myocardial wall stress and diastolic function as measured by the ratio of transmitral Doppler early filling velocity/tissue Doppler early diastolic mitral annular velocity (E/E′) and myocardial diastolic relaxation measured by tissue Doppler mitral annular motion (E′) in patients with hypertension in whom some degree of diastolic dysfunction is common but in whom EF and systolic function, as measured by conventional indices, is preserved. In a subsample of patients, we examined changes in EF1, TOR, E′, and E/E′ before and after the administration of nitroglycerin, a drug that influences ventricular dynamics^13,14^ and which is thought to decrease the TOR as do other nitric oxide donors.^15^

## Methods

### Study Population

Subjects (n=163, consecutively consenting subjects: 83 men, mean±SD age 46.5±16.5 years) were recruited from those evaluated for hypertension at Guy and St Thomas Hypertension Clinic. Subjects with valvular disease (more than mild regurgitation or stenosis), impaired LV systolic function (EF <50%), and arrhythmia other than sinus arrhythmia were excluded. The study was approved by the London Westminster Research Ethics Committee, and written informed consent was obtained. Anthropometric and clinical data were collected on a single study day and included medical and drug history, measurement of height and weight, measurement of peripheral and central blood pressure (BP) and other hemodynamic measures, echocardiography, and myocardial wall stress.

### BP, Aortic Root Pulse Wave Velocity, Reflection Coefficient, and Aortic Input Impedance

**Figure 1.**
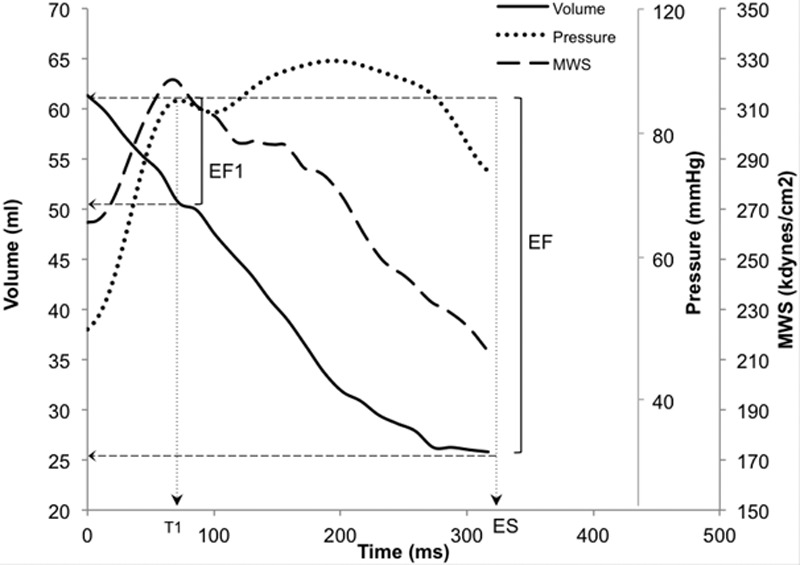
Endocardial volume curve (solid line) obtained by echo wall tracking, central aortic pressure waveform (dotted line) obtained by carotid tonometry, together with ejection-phase myocardial wall stress (dashed line), computed from the central aortic systolic pressure waveform and instantaneous left ventricular dimensions in a normotensive subject. Myocardial wall stress starts to fall at the first peak of central aortic pressure (T1), coinciding with peak aortic flow and maximal rate of ventricular shortening. First-phase ejection fraction (EF1) is percentage volume change between end diastole and T1. EF indicates ejection fraction; and MWS, myocardial wall stress.

Peripheral brachial BP was measured in a seated position with a Omron HEM 705-CP semiautomatic oscillometric recorder using the mean of 3 values of peripheral systolic BP and diastolic BP (DBP). Carotid arterial pressure waveforms were obtained by tonometry and calibrated from peripheral BP as detailed in the online-only Data Supplement. The time to first systolic shoulder of the central pressure waveform (T1, Figure 1), which closely approximates the time of maximal aortic flow and velocity of fiber shortening,^7^ was taken from the carotid pressure waveform. Aortic root pulse wave velocity (arPWV), reflection coefficient, calculated from the ratio of amplitudes of backward to forward pressure waves, aortic input impedance, and aortic characteristic impedance (Zc) were calculated from carotid pressure and aortic root flow velocities as detailed in the online-only Data Supplement.

### Echocardiography, Time-Resolved LV Dimensions, and EF1

A transthoracic echocardiographic study was obtained using the GE Vivid 7 ultrasound system (GE Healthcare, Little Chalfont, United Kingdom) and analyzed by 1 author (H.G.) while blinded to relevant clinical information. All echocardiographic views and measurements were performed using standard techniques according to the recommendations of the American Society of Echocardiography.^16^ Frequency and frame rate were optimized to allow adequate penetration for endocardial and epicardial border definition. LV mass was measured by 2-dimensional (2D) M-mode according to the American Society of Echocardiography recommendations.^16^ LV mass index was calculated by dividing LV mass by body surface area. Left atrial volume was measured by tracing the left atrial border from apical 4- and 2-chamber views. Pulsed wave Doppler was obtained using a 5-mm sample volume at the tips of the mitral leaflets in a 4-chamber view with a sweep speed of 150 mm/s. Tissue Doppler measures were obtained at levels of the lateral and septal mitral annulus to obtain an optimal spectral Doppler waveform. The E/E′ ratio was then calculated as a measure of diastolic function from the ratio of the transmitral Doppler E wave velocity to the mean of basal lateral and septal tissue Doppler E′ waves. Aortic flow velocity was obtained via pulsed wave Doppler distal to the aortic valve from an apical 5-chamber view. Global longitudinal strain (GLS) was calculated by placing 6 points along the endocardial border and adjusting the width of interest to accommodate the myocardial thickness, using a GE EchoPAC analysis package (GE Healthcare) from apical 4-, 2-, and 3-chamber views.

Time-resolved cavity volume and myocardial wall volume were obtained using a Tomtec analysis package (2D cardiac performance analysis; Tomtec, Munich, Germany) from a 2D apical 4-chamber view with optimized gain and depth using both endocardial and epicardial definitions (see online-only Data Supplement). The endocardium was initially defined by placing at least 6 points along it; the width of interest was then adjusted to accommodate myocardial thickness in each frame. Both the endocardial and epicardial border were then tracked automatically throughout the whole cardiac cycle. Auto-tracking was reviewed and edited frame by frame to ensure accurate tracking. End-diastolic volume (EDV) and end-systolic volume were derived from the LV volume curve. EF was calculated as the percentage change of LV volume from end diastole to end systole.

EF1 was introduced as a measure of early systolic ventricular function. EF1 is the percentage change in LV volume from end diastole to time T1 on the central aortic pressure waveform (Figure 1), a time that approximates the time of peak ventricular fiber shortening. EF1 is thus given as follows:


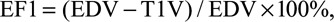
(1)

where EDV is end-diastolic endocardial volume, and T1V is endocardial volume at T1. We also calculated a previously described measure of early ejection, the first-third EF:


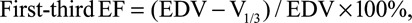
(2)

where EDV is end-diastolic endocardial volume, and V_1/3_ is endocardial volume at the time of the first third of ejection duration.^17^ Times of aortic valve opening and aortic valve closure were obtained from aortic valve spectral Doppler. Ejection duration was then defined from the beginning of aortic valve opening to aortic valve closure.

### Time-Varying Myocardial Wall Stress

Carotid applanation tonometry and echocardiography with wall tracking (as above) were used to obtain LV pressure and LV wall and cavity volume, respectively, over time during systole and hence to obtain time-resolved systolic myocardial wall stress (MWS) from the Arts formula as previously described by Chirinos et al^7^ and detailed in the online-only Data Supplement, TOR of MWS was defined as the time from the start of systole to peak MWS and expressed as a percentage of ejection duration. MWS time integral (area under the curve) in the first half (early phase) and second half (late phase) were computed and the ratio of late-/early-phase MWS calculated to assess the ventricular loading sequence.^18^

### Systolic and Diastolic Function in Response to Systemic and Intracoronary Nitroglycerin

Measurements of BP, arPWV, ventricular dynamics, and wall stress described above were repeated in a subsample of 18 subjects (14 men, aged 43.0±11.9 years). Measurements were performed at baseline and then between ≈5 and 15 minutes after 400 µg of nitroglycerin (delivered sublingually when hemodynamic measurements were stable). We also analyzed aortic root pressure and flow data obtained in a previous study^13^ before and during intracoronary infusion of nitroglycerin (1 µg/min, a dose below the threshold that causes systemic effects and which does not influence aortic input impedance) into the left coronary ostium in 10 hypertensive subjects with noncritical coronary heart disease and preserved LV function (mean±SD aged 57±17 years, aortic BP 139±39/69±8.3 mm Hg). We integrated flow velocity from end diastole to T1 and divided this by the integral over the whole of systole to obtain EF1/EF, assuming aortic cross-sectional area to remain constant over systole.

### Statistical Analysis

Characteristics are summarized as means±SD and results as means±SEM. To examine the relationship between ventricular ejection and duration of contractility (EF1 and TOR) with E/E′, subjects were divided into 3 groups using previously defined thresholds^19^ that corresponded to approximate tertiles of the distribution of E/E′: group 1 (E/E′<6.43), group 2 (E/E′=6.44–9.18), and group 3 (E/E′>9.18). Comparisons between these groups were then made using ANOVA with adjustment for confounding factors. Multiple regression analysis was used to examine the relationships between EF1 and TOR with E/E′ and E′, treating E/E′ and E′ as continuous variables. The following variables were forced into the multivariate models: age, sex, body mass index, antihypertensive medication, systolic BP, DBP, heart rate and T1, LV EDV, arPWV, and AV peak flow. In addition, the analysis was repeated using mean arterial pressure rather than systolic BP and DBP and using backward stepwise regression. Goodness of fit was expressed as the adjusted *r*^2^. Changes from baseline in hemodynamic measures after nitroglycerin were compared using Student paired *t* test. A *P* value <0.05 was considered statistically significant, and all tests were 2-tailed. Statistical analyses were performed using SPSS (SPSS Inc, Chicago, IL; version 21).

## Results

### Subject Characteristics

Subject characteristics in groups defined by E/E′ are shown in Table 1. Subjects with higher E/E′ were older than those with lower E/E′, and there were proportionally more women in groups with higher E/E′ compared with lower E/E′. Those with higher E/E′ also had higher BP, higher LV mass index, and larger left atrial volume and were more likely to be taking antihypertensive medications. These subjects also had higher arPWV and a trend toward higher Zc (Table 1, Figure S1 in the online-only Data Supplement). All subjects had preserved LV systolic function (EF>50%). There was no significant difference in heart rate, EF, or GLS across groups with varying E/E′.

**Table 1.**
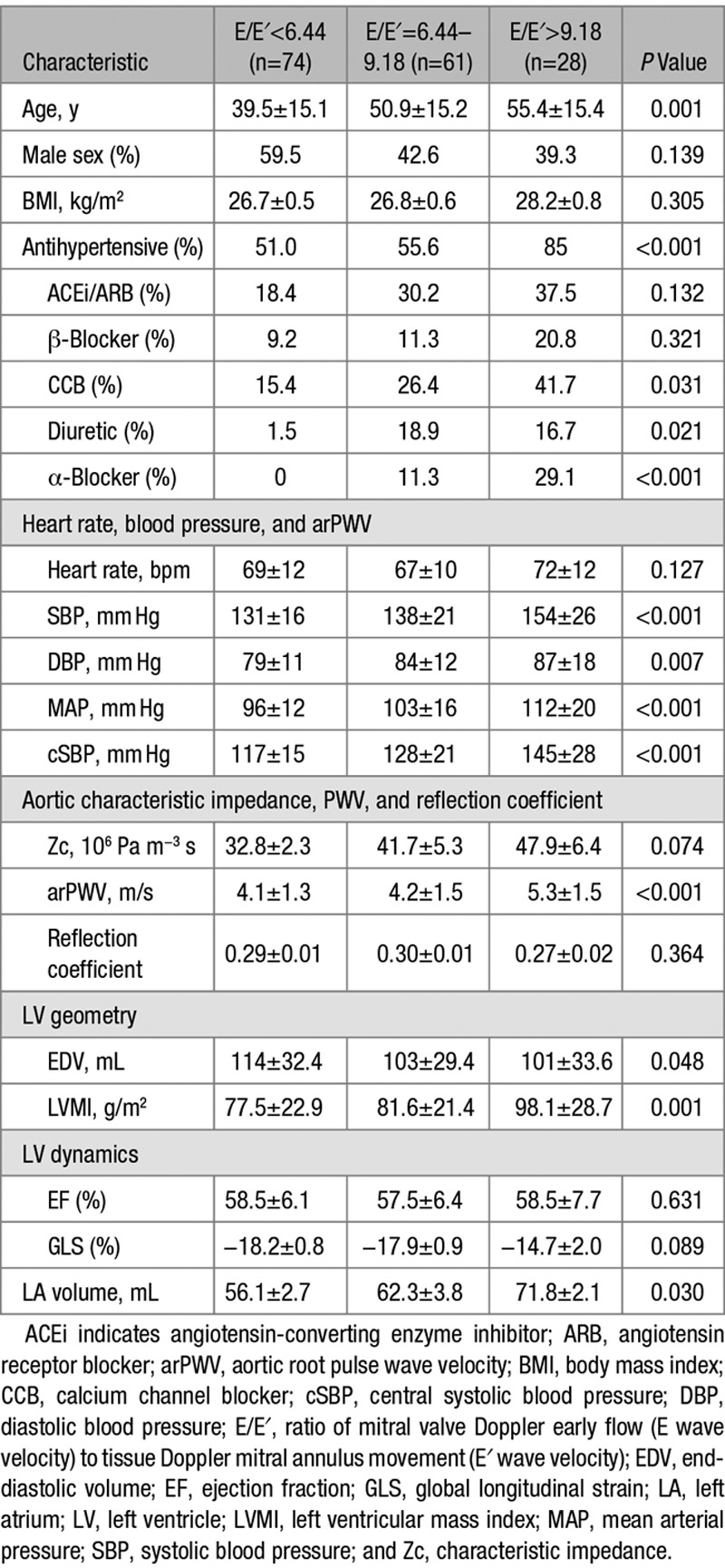
Subject Characteristics According to E/E′

### EF1 and Diastolic Function

**Figure 2.**
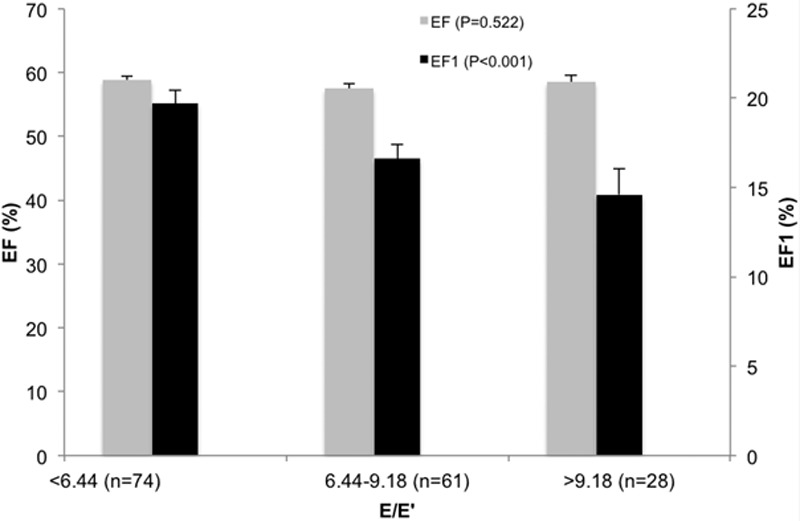
Total systolic ejection fraction (EF, gray bars) and first-phase ejection fraction (EF1, black bars; Figure 1) in 3 groups defined according to E/E′ (ratio of mitral valve Doppler early flow [E wave velocity] to tissue Doppler mitral annulus movement [E′ wave velocity]). Total EF was preserved and was similar between groups. EF1 was significantly lower in subjects with impaired diastolic function compared with those with preserved diastolic function.

Although overall EF (and also GLS) was similar across groups with increasing E/E′, EF1 was lower in subjects with higher E/E′ (Figure 2). In subjects with E/E′ >9.19, EF1 was 14.6±5.6% compared with 19.7±6.2% in those with E/E′ <6.44 (*P*<0.001, irrespective of adjustment for age and sex or other covariates), that is, a 26% difference in EF1 between these groups. T1, the time of measurement of EF1, was similar across the groups, and, therefore, this difference in EF1 represented a difference in rate of change of volume from the start of systole to T1. In multivariate regression (with all covariates forced into the model), EF1 was most strongly correlated with E/E′ (β=−0.34; *P*<0.001; Table 2) and E′ (β=0.48; *P*<0.001; substituting E′ for E/E′ and adjusting for the same covariates as in Table 2). On stepwise regression, it was equally strongly correlated with E/E′ and E′, but less strongly or weakly correlated with sex and DBP (or mean arterial pressure when this was substituted for systolic BP and DBP) and use of antihypertensive medication (Table 2). EF1 was not correlated with arPWV nor with reflection index. Repeating the analysis with Zc entered into the model showed no independent correlation of EF1 with Zc and no influence of Zc on the correlation of EF1 with E/E′ or E′. When EF1 was assessed for predictive power of E/E′ and E′ (Table S1), EF1 was the single most important predictor of E/E′ (β=−0.374; *P*=0.001) and E′ (β=0.380; *P*<0.001). When the ratio EF1/EF was substituted for EF1, we observed a similarly strong relationship between EF1/EF and E/E′ and between EF1/EF and TOR. However, there was no significant relationship between GLS and E/E′ nor between GLS and TOR. First-third EF was significantly associated with E/E′ (but less strongly than EF1; β=−0.211; *P*=0.048) and, when assessed for predictive power for E/E′ or E′, was displaced by EF1 (Table S1).

**Table 2.**
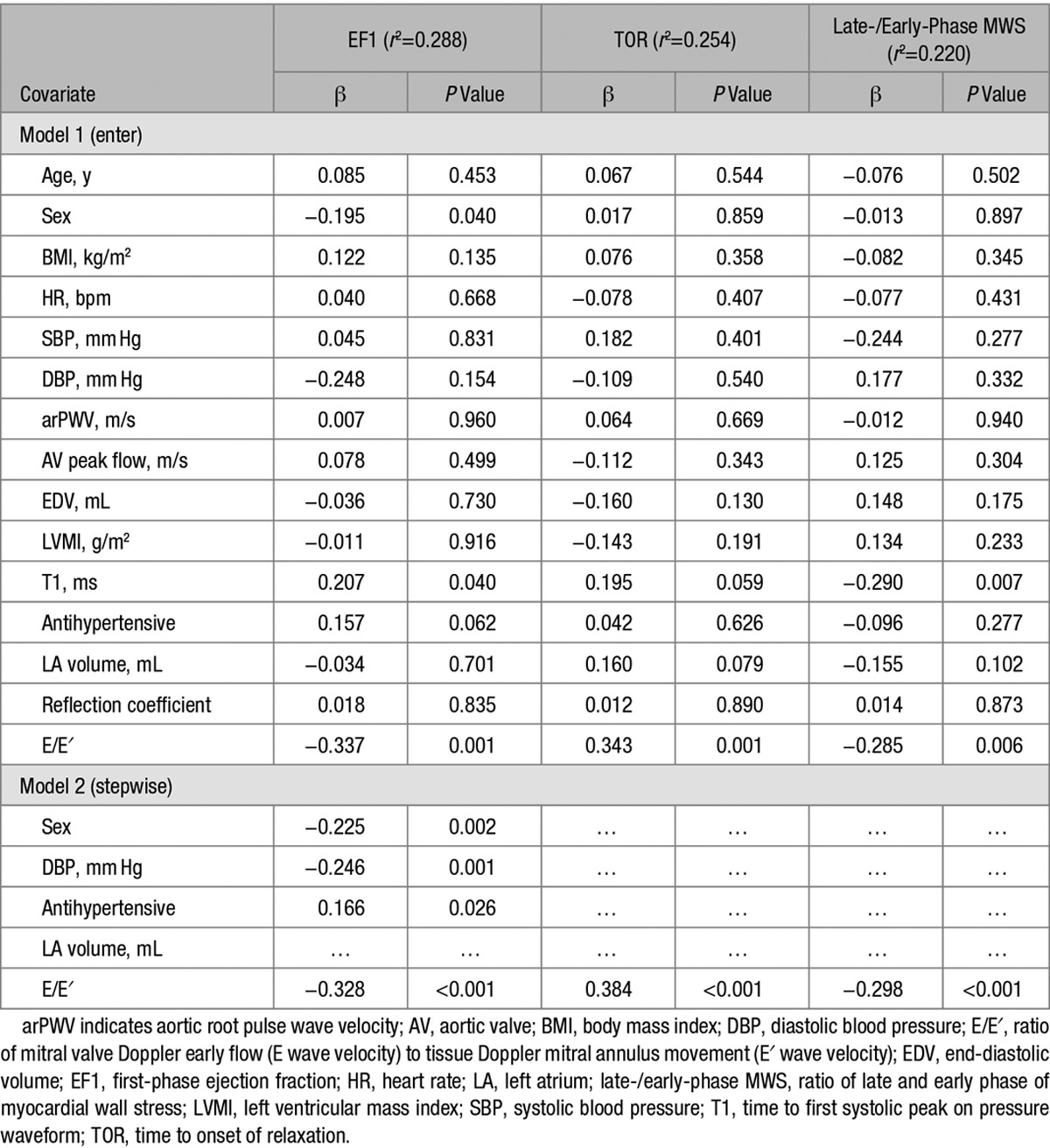
Multivariate Analysis of Relations Between EF1, TOR, and Late-/Early-Phase MWS With Hemodynamic and Echocardiographic Measures

### Duration of Myocardial Contraction and Diastolic Function

**Figure 3.**
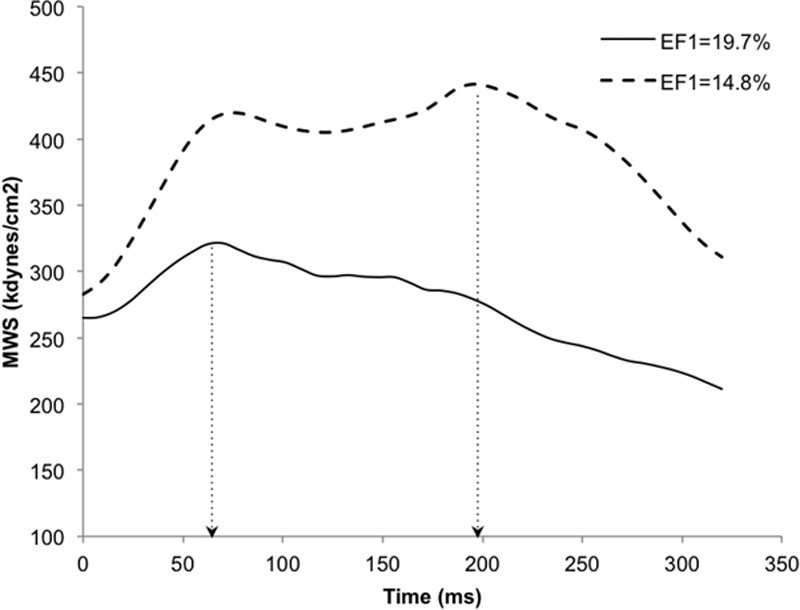
Typical myocardial wall stress traces in a subject with preserved systolic function and first-phase ejection fraction (EF1; solid line, E/E′=8.0, EF1=19.7%) and a subject with impaired diastolic function and reduced EF1 (dashed line, E/E′=16.6, EF1=14.8%) demonstrating longer time to onset of relaxation (TOR, dotted arrows) in the patient with diastolic dysfunction (TOR, 61.2% vs 22.0% of ejection duration). Both subjects had preserved ejection fraction (EF; 63.4% and 63.5%) and similar resting heart rate. E/E′ indicates ratio of mitral valve Doppler early flow (E wave velocity) to tissue Doppler mitral annulus movement (E′ wave velocity; and MWS, myocardial wall stress.

Myocardial contraction was prolonged with greater TOR in subjects with higher E/E′ compared with lower E/E′ (Figure 3; Figures S2 and S3). This was because of an early fall in MWS after T1 in subjects with preserved EF1 and preserved diastolic function, with a dissociation between MWS and pressure, but a closer relationship between pressure and MWS, with MWS prolonged toward the second pressure peak in subjects with reduced EF1 and diastolic dysfunction (Figure S3). In subjects with E/E′ >9.19, TOR was 38±3% compared with 27±1% in those with E/E′ <6.43 (*P*<0.001, irrespective of adjustment for age and sex or other covariates). On multivariate analysis, TOR was positively associated with E/E′ (β=0.34; *P*<0.001; Table 2) and E′ (β=−0.338; *P*=0.006; Table 2). These results were unchanged irrespective of whether TOR was expressed as a time or % of ejection duration. Late-/early-phase MWS ratio was higher in subjects with higher E/E′ compared with lower E/E′. In subjects with E/E′ >9.19, late-/early-phase MWS ratio was 0.82 compared with 0.91 in those with E/E′ <6.43 (*P*<0.001, irrespective of adjustment for age and sex or other covariates). On multivariate analysis, late-/early-phase MWS ratio was positively associated with E/E′ (β=0.231; *P*=0.024; Table 2).

### Effect of Systemic and Intracoronary Nitroglycerin on Systolic and Diastolic Function

Systemic nitroglycerin produced a modest but significant reduction in BP (Table 3). It had no significant effect on EF but increased EF1 significantly from 15.2±1.8% to 20.3±2.6% (*P*<0.001), reduced TOR from 41.7±3.6% to 32.5±1.7% (*P*<0.05), increased E′ from 8.5±0.8 to 10.0±0.9 cm/s (*P*=0.047), and reduced E/E′ from 8.5±0.6 to 6.6±0.5 (*P*=0.014). Typical wall stress traces before and after nitroglycerin are shown in Figure S4. Intracoronary nitroglycerin produced a significant increase in EF1/EF from 21.8±0.12% to 35.4±0.15% (*P*=0.005).

**Table 3.**
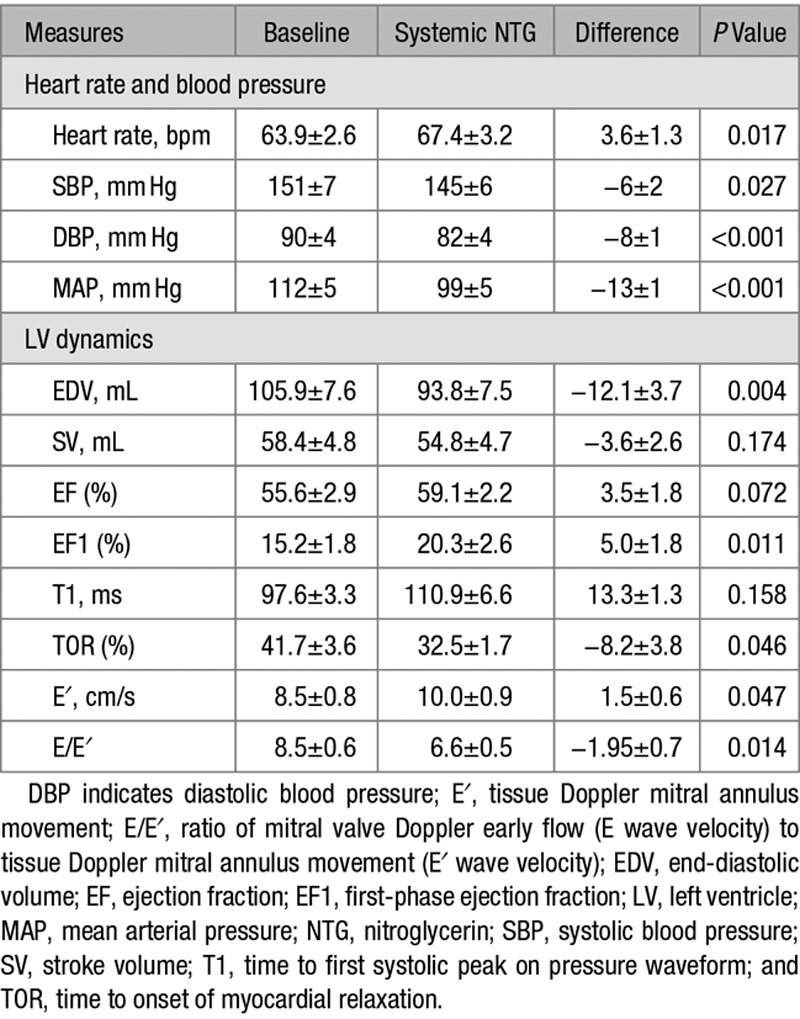
Effects of Nitroglycerin on Blood Pressure, Systolic, and Diastolic Function

## Discussion

Shortening deactivation of cardiac muscle contraction in vivo is likely to involve a transduction of shortening that occurs early in systole at or before T1 because, in healthy subjects, the myocardium starts to relax from this point onward.^7^ The major novelty of the present work is the use of a measure of early shortening that triggers deactivation such as EF1, which, if impaired, would be expected to lead to sustained contraction that may compromise diastolic function. The main experimental findings are that, in hypertensive subjects with hypertension of varying severity and with varying structural remodeling, there is a relationship between EF1, temporal patterns of systolic stress, and myocardial relaxation assessed by E′ and diastolic function assessed by E/E′, a measure of diastolic function that is one of the best predictors of outcome in hypertension.^19^ These findings with regard systolic stress are similar to those from the Asklepios population (a predominantly normotensive population) in which delayed systolic stress was predictive of impaired diastolic relaxation.^18^ This suggests that the link between the distribution of stress over systole and diastolic dysfunction is likely to extend from the normotensive to hypertensive population.

Diastolic and systolic dysfunction and mortality are thought to be related to arterial wave reflections in hypertension,^20–22^ and the lower EF1 we observed could be related to reflections and other characteristics of afterload. EF1 and TOR were related to E/E′ and E′ independent of ventricular cavity and wall dimensions and of afterload as assessed by BP, arPWV (which captured the main difference in impedance between groups with differing E/E′), and late systolic reflection as measured by the ratio of the amplitude of the backward/forward pressure wave. Thus, if afterload is the primary determinant of diastolic function, the effects of afterload are likely mediated through EF1. However, the stronger relationship between diastolic function and EF1 than between diastolic function and measures of afterload suggests that, regardless of the cause, EF1 is more directly/immediately related to diastolic dysfunction, with early generation of wall stress and shortening leading to early relaxation and preserved diastolic function. Chirinos et al^7^ found concentric remodeling of the LV to be associated with a less efficient pressure–stress midsystolic shift which favored increased late systolic wall stress, and this could be a mechanism through which sustained afterload leads to an alteration in early systolic function.

Associations of EF1 with diastolic function could be secondary to structural changes such as interstitial fibrosis. However, our studies with nitroglycerin show that an increase in EF1 is associated with a reduction in TOR and improved diastolic function, demonstrating that diastolic function can be modified and supporting a causal effect of EF1 on subsequent contraction/relaxation dynamics. To what degree changes in EF1 were because of a reduction in preload/afterload or to a direct action of nitroglycerin on the myocardium cannot be determined from the systemic administration of nitroglycerin. However, in our analysis of previous intracoronary nitroglycerin data, we observed an increase in EF1/EF, when there is no significant change in afterload. This, together with previous studies showing that intracoronary infusion of nitric oxide donors decreases the TOR and improves diastolic relaxation,^15^ suggests the effect of nitroglycerin is mediated, at least in part, by a direct effect of nitroglycerin on the myocardium and a reduction in preload.

Although diastolic dysfunction with preserved overall EF is recognized as a distinct phenotype,^23^ it is also accepted that diastolic dysfunction often coexists with systolic dysfunction, particularly systolic dysfunction that may not manifest as a reduction in overall EF.^24^ This study supports this view with an emphasis on EF1, representing the extent of early myocyte shortening, as a potential primary determinant of subsequent events in systole and diastole. It demonstrates that profound degrees of early systolic dysfunction resulting in a reduction in EF1 of >25% are seen in the absence of any change in overall EF. The sustained contraction that is seen in association with a reduced EF1 may represent a compensatory mechanism to maintain the overall EF, so that this is preserved even in the face of marked early systolic dysfunction. A link between early systolic and diastolic dysfunction mediated by a reverse of the shortening deactivation phenomenon would explain why E/E′ is prolonged after myocardial infarction and after angioplasty and is a good predictor of outcome after events such as these^25,26^ that would not necessarily be expected to impact on diastolic relaxation per se.

Our study is subject to several important limitations. First, our conclusions relate only to hypertensive subjects. We studied these because of the importance of hypertension as a risk factor for heart failure with preserved EF and relatively modest departure from normal physiology. Further studies will be required to establish the nature of the link between early systolic dysfunction, sustained myocardial contraction, and impaired diastolic relaxation in cardiomyopathies of differing etiologies and severity, particularly those with preserved EF. Conclusions on causality cannot be drawn from the cross-sectional observations, although effects of nitroglycerin and biological plausibility support a causal role of EF1. We used noninvasive estimates of LV pressure and wall stress that are limited by the calibration of noninvasive BP. However, although calibration errors might influence absolute values, they are less likely to influence the relative timing of wall stress. Our measurement of reflection coefficient may be limited by rectified reflections.^27^ Doppler flow velocity measurements are subject to variation because of probe position which could have confounded our measurements of impedance, although such variation would likely be randomly distributed across the comparator groups. Our measures of ventricular volumes were obtained from a single plane across the ventricle and thus provide a 2D measure of shortening. Three-dimensional imaging will be required to determine whether alternative measures perform similarly and to assess first-phase ejection in subjects with regional wall abnormalities. Although diastolic function could relate to total circumferential or longitudinal strain^28^ which, because of compensatory changes may be abnormal in hypertension despite preserved overall EF, we found no significant relationship between E/E′ and GLS, a more sensitive measure of impaired total shortening than overall EF.^29^ This underlines the importance of early rather than total shortening as a potential determinant of diastolic function at least as measured by E/E′. Although we observed a stronger relationship between EF1 and E/E′ than between first-third EF and E/E′, further studies will be required to establish whether EF1 is a better measure of shortening deactivation than other older measures such as first-third EF and to determine the features of diastolic function (other than E′ and E/E′) that are most closely associated with early systolic dysfunction.

## Perspectives

A stress- or length-dependent locking of myosin motors onto the thick filament of the cardiac myocyte, a length-dependent reduction in myofibrillar calcium sensitivity, and a velocity-dependent reduction of myosin crossbridge attachment may all play a part in shortening deactivation, whereby shortening and early ejection from the LV leads to a rapid reduction in myocardial wall stress. Our findings that, in hypertensive patients, impaired shortening as measured by EF1 is a predictor of diastolic dysfunction and can be modified by nitroglycerin provide a potential mechanistic link between early systolic dysfunction, sustained myocardial contraction, and impaired diastolic relaxation.

In conclusion, in hypertensive patients, a reduced early systolic EF correlates with sustained myocardial stress in later systole and with diastolic dysfunction. EF1, which we suggest is linked to subsequent contraction/relaxation through the shortening deactivation phenomenon, may be an important diagnostic measurement and therapeutic target to prevent progression to heart failure, particularly that which is associated with hypertension and preserved EF.

## Sources of Funding

This research was supported by the British Heart Foundation. We acknowledge financial support from the Department of Health via the National Institute for Health Research (NIHR) comprehensive Biomedical Research Centre and Clinical Research Facilities awards to Guy and St Thomas NHS Foundation Trust in partnership with King’s College London and King’s College Hospital NHS Foundation Trust.

## Disclosures

H. Gu and P.J. Chowienczyk are named on a patent application relating to early phase ejection fraction.

## Supplementary Material

## Novelty and Significance

### What Is New?

- Shortening deactivation provides a unifying concept linking systolic to diastolic dysfunction. To quantify early systolic shortening, we introduced first-phase ejection fraction (EF1), the ejection fraction up to the time of maximal ventricular fiber shortening. In regression analysis in hypertensive patients with varying degrees of diastolic function, incorporating measures of afterload and echocardiographic measures of ventricular structure and function, EF1 was found to be the best predictor of diastolic function and relaxation as measured by the ratio of transmitral Doppler early filling velocity to tissue Doppler early diastolic mitral annular velocity (E/E′) and E′. EF1 could be modulated independent of afterload by nitroglycerin with subsequent improvement in diastolic function.

### What Is Relevant?

- In hypertensive patients with preclinical diastolic dysfunction and preserved overall ejection fraction, EF1 is impaired and associated with sustained myocardial contraction and diastolic dysfunction. Therapeutic interventions that improve first-phase ejection might improve diastolic function.

### Summary

In hypertensive patients, reduced early systolic ejection fraction is associated with sustained contraction and diastolic dysfunction. EF1, which we suggest is linked to subsequent contraction/relaxation through the shortening deactivation phenomenon, may be an important diagnostic measurement and therapeutic target to prevent progression to heart failure particularly that is associated with hypertension and preserved ejection fraction.
