# Supplementary material for: Reduced First-Phase Ejection Fraction and Sustained Myocardial Wall Stress in Hypertensive Patients With Diastolic Dysfunction: A Manifestation of Impaired Shortening Deactivation That Links Systolic to Diastolic Dysfunction and Preserves Systolic Ejection Fraction
Source: Hypertension. 2017 Mar 8;69(4):633–40. doi: 10.1161/HYPERTENSIONAHA.116.08545 (PMC5344180; doi:10.1161/HYPERTENSIONAHA.116.08545)
Supplement: Supplementary file 1 [file hyp-69-633-s001.docx]

**Reduced first-phase ejection fraction and sustained myocardial wall stress in hypertensive patients with diastolic dysfunction**

Supplement Methods and Data

Haotian Gu*

Ye Li*

Henry Fok*

John Simpson†

Jonathan C Kentish*

Ajay Shah*

Phil Chowienczyk*

* King’s College London, British Heart Foundation Centre of Research Excellence, London, UK

† Department of Congenital Heart Disease, Evelina London Children’s Hospital, UK

Correspondence:

Phil Chowienczyk, Clinical Pharmacology, St Thomas’ Hospital, London, UK, SE1 7EH; Tel: 0044-2071884799; Fax: 0044-2071885116;

Email: [phil.chowienczyk@kcl.ac.uk](mailto:phil.chowienczyk@kcl.ac.uk)

**Central blood pressure, aortic root pulse wave velocity, reflection coefficient and aortic input impedance**

Carotid and radial arterial pressure waveforms were obtained by a high-fidelity micromanometer (SPC-301; Millar Instruments, Houston, TX) from the left radial and left carotid artery and processed by the SphygmoCor device by an experienced operator (Atcor medical, Australia). Radial and carotid waveforms meeting the inbuilt quality control criteria of the SphygmoCor device (operator index > 75%) were averaged and calibrated from peripheral brachial measures of systolic (SBP) and diastolic blood pressure (DBP), from which mean arterial pressure (MAP) was calculated by integrating the radial waveform. The carotid pressure waveform was then calibrated from MAP and peripheral DBP which (unlike SBP) are equal at aortic and peripheral sites ^1^. Ensemble averaged carotid pressure was used as surrogate for ascending aortic pressure ^2^. Previous studies have shown that, because of its close proximity to the aorta, the carotid waveform can be used as a surrogate of the central aortic pressure waveform and left ventricular pressure during systole ^2^. The carotid waveform was used to identify the time to first systolic shoulder of the central pressure waveform (T1), which closely approximates the time of maximal aortic flow and ventricular shortening ^3^.

An aortic flow waveform was obtained from maximal aortic flow velocity (obtained in the left ventricular outflow tract (LVOT) using pulsed wave Doppler from 5-chamber view) multiplied by the LVOT cross-sectional area (Internal diameter of the LVOT was measured in the parasternal long-axis view at the valve annulus, and LVOT area was calculated assuming circularity).^4^ Pressure and flow were decomposed into a series of sinusoidal harmonics, each represented by its amplitude (modulus) and phase angle. Input impedance was calculated as the ratio of the pressure and flow harmonics over the frequency range 0 – 10 Hz. Characteristic impedance (Zc) was calculated as the mean of the modulus of input impedance over the frequency range 3 to 10 Hz.^4^ Aortic root pulse wave velocity (arPWV), was calculated from the relationship between pressure and flow velocity during initial ejection (before T1) by the single-point technique^5^ (the sum of squares method), using the following equation:

$$arPWV=\frac{1}{\rho}\sqrt{\frac{\sum dP^{2}}{\sum{dU}^{2}}}$$

where ρ is the density of blood, P is central aortic pressure (derived from carotid pressure by SphygmoCor) and U is aortic flow velocity (derived from echocardiography pulsed-wave Doppler in the aortic root from an apical 5-chamber view). arPWV is related to characteristic impedance (Zc, measured in dyne.s.cm^-5^) and aortic root cross-sectional area (A) by: arPWV=Zc.A/ ρ.^6^

Forward and backward pressure waveform wave decomposition was performed based on the conservation of mass and momentum and using Parker’s time domain approach^7^ to obtain forward (P_f_) and backward (P_b_) pressure wave components of pressure (P) so that: P_f_ + P_b_ = P – P_d_ where P is total pressure and P_d_ is the diastolic pressure. P_f_ and P_b_ are given by:

$$P_{f}=\frac{1}{2}\sum[(dP+\rho cdU)]$$

$$P_{b}=\frac{1}{2}\sum[(dP-\rho cdU)]$$

Where U is flow velocity, ρ is blood density, and c is pulse wave velocity which was calculated using the method of the sum of squares. The reflection coefficient (R=Pb/Pf) was calculated as the ratio of the amplitude (peak) of the backward wave to that of the forward wave.

**Time-varying myocardial wall stress**

Ejection-phase time-varying myocardial wall stress was computed according to Arts:^8^

MWS=P/[1/3ln(1+Vw/Vc)]

Where P=LV pressure, ln=natural log, Vw=myocardial wall volume, and Vc=LV cavity volume. Myocardial wall volume was calculated by deduction of LV cavity volume from LV epicardial volume at each time point. Cavity and epicardial volume tracking were gated by R-R interval on ECG. Isovolumetric contraction period was measured from on set of R wave to AVO, and end of systole was defined as R wave to AVC.

**Reproducibility assessment for LV volumes and EF1**

The reproducibility of this wall tracking for EDV and ESV were evaluated from measurements on 12 subjects, repeated on two separate occasions separated by approximately 3 months by the same observer and by two independent observers on the same occasion. The between-visit coefficient of variation (CV, equal to the SD as a percentage of the mean) was 2.0%, 3.1% and 4.3% for EDV, ESV and EF1 respectively. Inter-observer CVs were 3.0% for ED and ES cavity volumes respectively.

**Reference**

1. Pauca AL, Wallenhaupt SL, Kon ND, Tucker WY. Does radial artery pressure accurately reflect aortic pressure? *Chest.* 1992;102:1193-1198.

2. Chen CH, Ting CT, Nussbacher A, Nevo E, Kass DA, Pak P, Wang SP, Chang MS, Yin FC. Validation of carotid artery tonometry as a means of estimating augmentation index of ascending aortic pressure. *Hypertension.* 1996;27:168-175.

3. Chirinos JA, Segers P, Gupta AK, Swillens A, Rietzschel ER, De Buyzere ML, Kirkpatrick JN, Gillebert TC, Wang Y, Keane MG, Townsend R, Ferrari VA, Wiegers SE, St John Sutton M. Time-varying myocardial stress and systolic pressure-stress relationship: role in myocardial-arterial coupling in hypertension. *Circulation.* 2009;119:2798-2807.

4. Segers P, Rietzschel ER, De Buyzere ML, Vermeersch SJ, De BD, Van Bortel LM, De BG, Gillebert TC, Verdonck PR. Noninvasive (input) impedance, pulse wave velocity, and wave reflection in healthy middle-aged men and women. *Hypertension.* 2007;49:1248-1255.

5. Davies JE, Whinnett ZI, Francis DP, Willson K, Foale RA, Malik IS, Hughes AD, Parker KH, Mayet J. Use of simultaneous pressure and velocity measurements to estimate arterial wave speed at a single site in humans. *Am J Physiol Heart Circ Physiol.* 2006;290:H878-885.

6. Nichols WW, O'Rourke MF. *McDonald's blood flow in arteries. Theoretical, experimental and clinical principles.* London: Arnold; 1998.

7. Parker KH, Jones CJ. Forward and backward running waves in the arteries: analysis using the method of characteristics. *J Biomech Eng.* 1990;112:322-326.

8. Arts T, Bovendeerd PH, Prinzen FW, Reneman RS. Relation between left ventricular cavity pressure and volume and systolic fiber stress and strain in the wall. *Biophys J.* 1991;59:93-102.

**Table S1.** EF1 as predictor of E/e’ and e’ in a multivariate model

|  | | | **E/e’**  **(r^2^=0.487)** | |  | **e’**  **(r^2^=0.559)** | |  |
| --- | --- | --- | --- | --- | --- | --- | --- | --- |
|  | **Covariate** | | **β** | **P Value** |  | **β** | **P Value** |  |
|  | **Model 1 (Enter)** | |  |  |  |  |  |  |
|  | | **Age (years)** | 0.225 | **0.013** |  | -0.515 | **<0.001** |  |
|  | | **Gender** | -0.033 | 0.687 |  | 0.180 | 0.014 |  |
|  | | **BMI (kg/m^2^)** | 0.041 | 0.562 |  | -0.094 | 0.133 |  |
|  | | **Anti-hypertensive** | 0.029 | 0.690 |  | -0.116 | 0.073 |  |
|  | | **HR (bpm)** | -0.104 | 0.316 |  | 0.128 | 0.160 |  |
|  | | **SBP (mmHg)** | 0.067 | 0.739 |  | 0.265 | 0.134 |  |
|  | | **DBP (mmHg)** | 0.083 | 0.591 |  | -0.423 | **0.002** |  |
|  | | **PWV (m/s)** | -0.161 | 0.125 |  | -0.223 | **0.016** |  |
|  | | **SVR (mmHg/ml)** | -0.186 | 0.181 |  | 0.014 | 0.908 |  |
|  | | **Pf (m/s)** | 0.331 | **0.027** |  | 0.123 | 0.343 |  |
|  | | **Pb (m/s)** | 0.044 | 0.620 |  | 0.008 | 0.916 |  |
|  | | **EDV (ml)** | -0.394 | **0.025** |  | 0.185 | 0.171 |  |
|  | | **LVMI (g/m^2^)** | 0.235 | **0.010** |  | -0.047 | 0.553 |  |
|  | | **LA Volume (ml)** | 0.051 | 0.508 |  | -0.055 | 0.417 |  |
|  | | **First Third EF (%)** | 0.112 | 0.303 |  | -0.079 | 0.410 |  |
|  | | **EF1 (%)** | -0.374 | **0.001** |  | 0.380 | **<0.001** |  |

E/e’: ratio of mitral valve Doppler early flow (E wave velocity) to tissue Doppler mitral annulus movement (e’ wave velocity); e’: tissue Doppler mitral annulus movement; BMI: body mass index; HR: heart rate; bmp: beats per minute; SBP: systolic blood pressure; DBP: diastolic blood pressure; PWV: pulse wave velocity; EDV: end-diastolic volume; LVMI: left ventricular mass index; LA: left atrium; Pf: peak forward flow velocity; Pb: peak backward flow velocity; SVR: systemic vascular resistance; EF1: first-phase ejection fraction; First Third EF: First third ejection fraction.


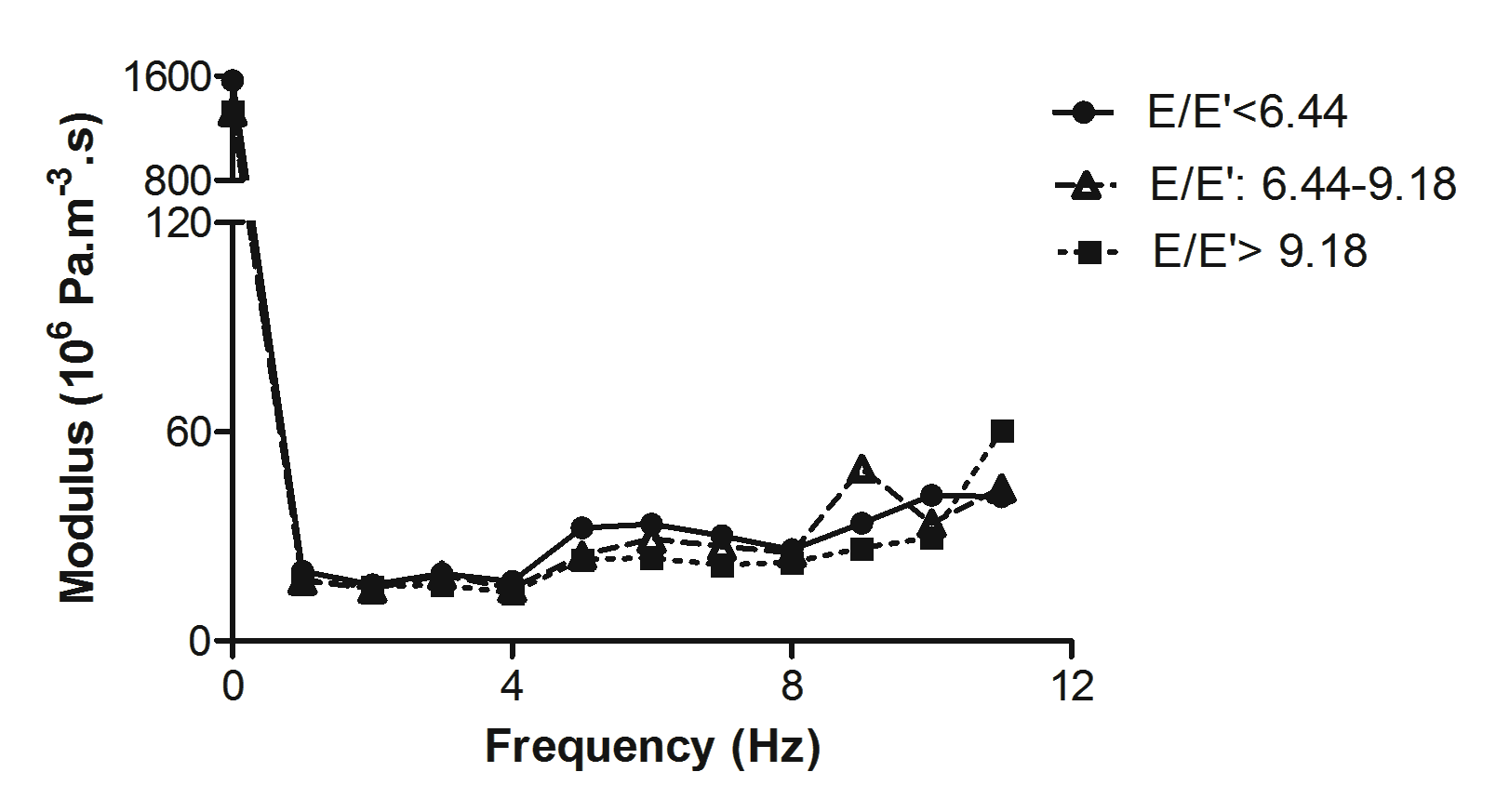


(a)


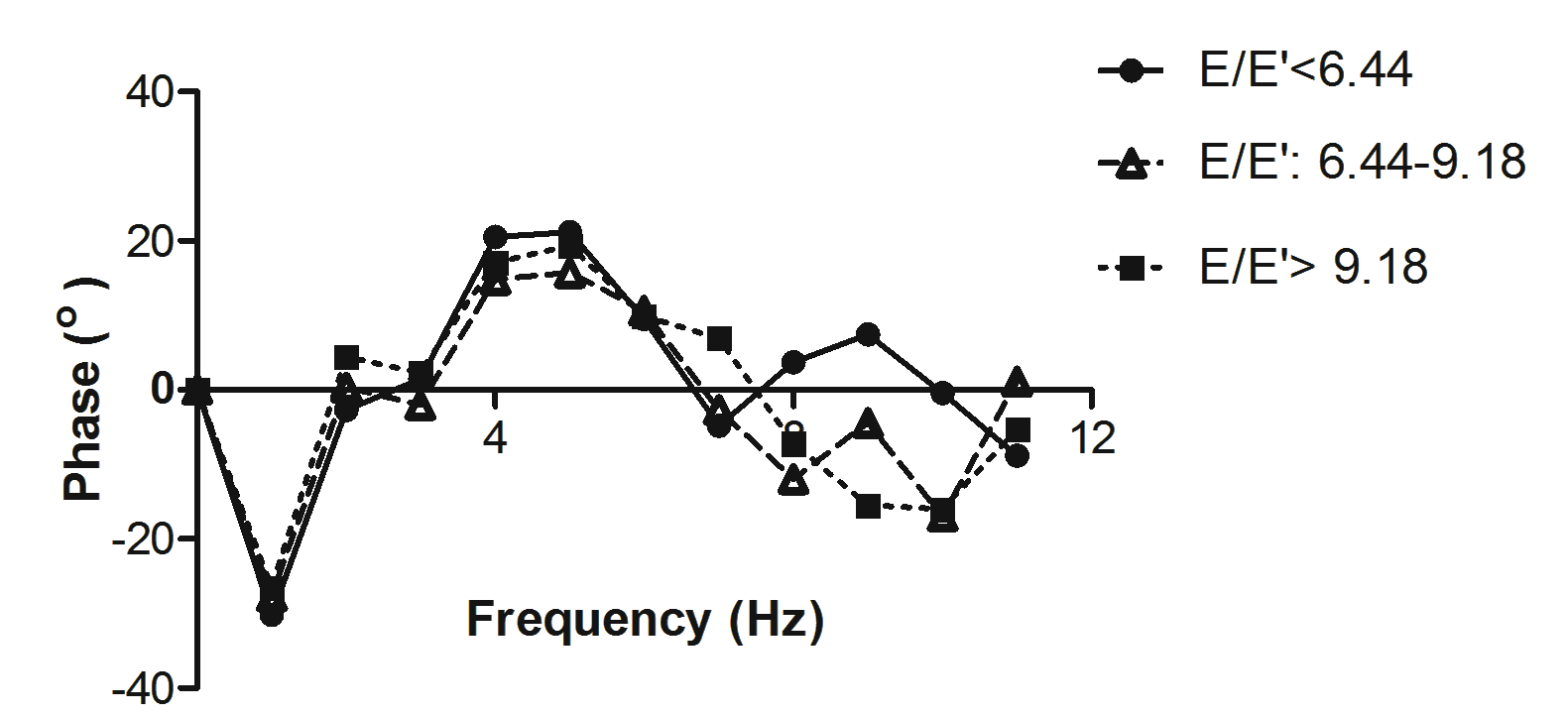


(b)

**Figure S1.** a) Average modulus and b) phase of aortic input impedance in three groups divided by E/e’. E/e’<6.44 (solid line), E/e’: 6.44-9.18 (dashed line) and E/e’ >9.18 (dotted line). Neither modulus nor phase are significantly different between groups.

**Figure S2.** Time from end-diastole to onset of myocardial relaxation (TOR) in three groups defined according to E/e’. Myocardial contraction was sustained to a greater extent in subjects with impaired diastolic function compared to those with preserved diastolic function with TOR higher in subjects with impaired diastolic function.

(a)

(b)

**Figure S3.** Typical central pressure (solid line) and ejection duration myocardial wall stress traces (dashed line) in (a), a subject with preserved systolic function and first-phase ejection fraction and (b), a subject with impaired diastolic function and reduced EF1.

**Figure S4.** Myocardial wall stress at baseline (solid line) and after administration of nitroglycerin (NTG, dashed line) in a 65 year-old man. After NTG, first-phase ejection fraction (EF1) increased from 11.6% to 17.9%, and time to onset of relaxation (TOR) reduced from 53 to 32% of ejection duration.
